# OmicsON – Integration of omics data with molecular networks and statistical procedures

**DOI:** 10.1371/journal.pone.0235398

**Published:** 2020-07-29

**Authors:** Cezary Turek, Sonia Wróbel, Monika Piwowar

**Affiliations:** 1 Department of Bioinformatics and Telemedicine, Jagiellonian University–Medical College, Krakow, Poland; 2 Department of Medical Physics, Jagiellonian University, Marian Smoluchowski Institute of Physics, Krakow, Poland; Instituto de Investigacion Sanitaria INCLIVA, SPAIN

## Abstract

A huge amount of atomized biological data collected in various databases and the need for a description of their relation by theoretical methods causes the development of data integration methods. The omics data analysis by integration of biological knowledge with mathematical procedures implemented in the OmicsON R library is presented in the paper. OmicsON is a tool for the integration of two sets of data: transcriptomics and metabolomics. In the workflow of the library, the functional grouping and statistical analysis are applied. Subgroups among the transcriptomic and metabolomics sets are created based on the biological knowledge stored in Reactome and String databases. It gives the possibility to analyze such sets of data by multivariate statistical procedures like Canonical Correlation Analysis (CCA) or Partial Least Squares (PLS). The integration of metabolomic and transcriptomic data based on the methodology contained in OmicsON helps to easily obtain information on the connection of data from two different sets. This information can significantly help in assessing the relationship between gene expression and metabolite concentrations, which in turn facilitates the biological interpretation of the analyzed process.

## Introduction

Technologies of molecular biology providing a big amount of data have given rise to large-scale biological datasets. Analysis of such data imposes methodological challenges relating to the complex structure and the size of the data. Driven by high-throughput omics technologies and the computational surge, it enables multi-scale and insightful overviews of cells, organisms, and populations. This approach has had a huge impact on the discovery of next-generation diagnostics, biomarkers, and drugs in the precision medicine era [1].

Systemic exploration of complex interactions in biological systems because of the development of new technologies and analytical methods allows the creation of clinically useful tools [2]. Despite their promise, the translation of these technologies into clinically actionable tools has been slow [3] [4] [5]. This is due to the complexity of the issues, but also with the problems arising from the data standardization, data sharing, storing Omics data appropriately and exploring Omics data [6]. Proper organizing and standardizing data, as well as newly developed methodologies and computational algorithms, lead to comprehensive multi-omics and clinical data integration thus enable insightful interpretation of biological processes [7] [8] [9] [10] [11] [12] [13].

In this paper, data analysis by integrative biological knowledge (from biological databases) with mathematical procedures (multidimensional statistical analysis) developed in the OmicsON library is presented. The procedure implemented in the OmicsON library is based on a previously developed and published algorithm [14].

## Design and implementation

The OmicsON library was written in R language [15] according to the Bioconductor guidelines. Particular steps analysis were described based on the lipidomics and transcriptomic data [16]. The standardization and evaluation, as well as the quality control of the data sets, is beyond the functionality of the OmicsON library. It is assumed that pre-processing data is done before analysis in OmicsON (because of different types of e.g. microarray technologies and data formats).

**Fig 1:**
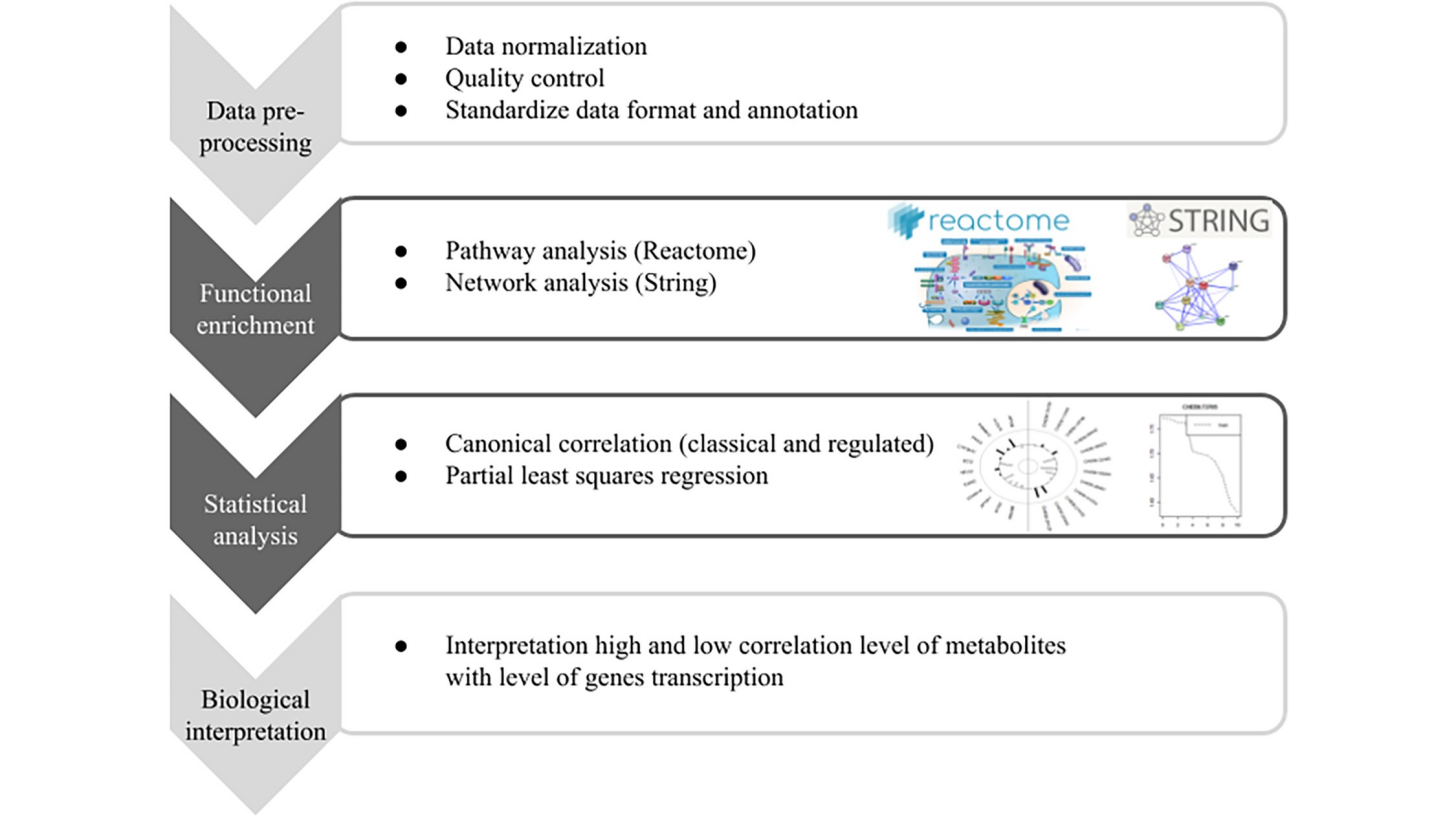
The workflow of lipidomics (metabolomics) and transcriptomics data integration. Light gray means steps performed outside and the dark gray steps performed using the OmicsON library.

The transcriptomics and lipidomics analysis based on the OmicsON library follow as on the diagram is presented (Fig 1). The workflow in OmicsON consists of a functional pathway and network analysis, statistical analysis by using Canonical Correlation Analysis and Partial Least Square regression. The significant associations between lipidomic and transcriptomic data are calculated. In this way, the researcher receives a set of information for substantive assessment.

### Data input

Input data is a set of transcriptomic and lipidomic data that were previously prepared, i.e. normalized, and on which quality control was carried out. Variable distributions should be multidimensional normal. As input, the library expects two matrices to be stored in a text file (tab-delimited): transcriptional and lipidomic sets (fatty acids). The transcriptomic set in the columns should contain the gene symbols according to the HGNC nomenclature [17] and in the lines the levels of gene expression for the individual samples. In the metabolomics dataset in the columns should be the metabolites identifiers according to the CHEBI nomenclature [18] and in the lines should be quantitative data, eg metabolite concentrations.

### Gene mapping and grouping data

Gene mapping is a step in which data are decorated by searching for ontologically related molecules present in Reactome’s pathways [19]. Results from the Reactome step are decorated by extra interactions of genes based on knowledge taken from the STRING [20] database. Information from the Reactome and the STRING databases gives the possibility for reducing the size dimension of big gene transcription datasets. Finally, it obtains a broad list of potential interactions to be sifted later by statistical testing with two goals: to minimize potential bias towards well-characterized biochemical pathways and, at the same time, to propose new putative links relevant for the specific study (tissue, conditions).

OmicsON allows the mapping of HGNC, Ensemble, and UniProt gene identifiers to find relationships in paths deposited in Reactome database (data sets in supplementary materials: S1 Table).

Enrichment of information on sets of genes associated with fatty acids can be performed for subsets of fatty acids (grouping data) [21]. Grouping data can be done before or/and after gene mapping. For example, the correlation analysis of many thousands of transcriptomic data (microarrays) among a few (or even several dozens) samples with metabolomics data are difficult to carry out and in many cases impossible based on known statistical techniques. In the case of classical canonical correlation analysis, the use of such a large matrix for correlation with a metabolic set is not feasible. In such cases, it is justified to divide the data into subgroups according to justified criteria.

The division into subsets of fatty acids and genes has not been automated in OmicsON. Anyone can make such a breakdown according to criteria that can be different depending on the analytical approach.

### Statistical methods

Subgroups of gene and metabolite sets are analyzed with Canonical Correlation (CCA) [22] and Partial Least Squares (PLS) [23] multivariate procedures. These two mathematical methods can be both used for the same datasets (subgroups) or can be used separately depending on the dimension of the datasets (PLS is free of dataset dimension limits).

#### Canonical Correlation Analysis (CCA)

The canonical analysis is a generalization of multiple regression (regression analysis of many independent variables {X1,…, Xk} into one dependent Y) into two sets of variables, i.e. a set of independent variables {X1,…, Xk} and a set of dependent variables {Y1,…, Ym}. CCA allows you to find the answers to the following questions:

- What is the influence of the group of independent variables on the group of effect variables (dependent)? In our case, independent variables {X1,…, Xk}—expressions for the group of dependent variables {Y1,…, Ym}—lipidomics.
- Is, and if so, which of the independent variables {X1,…, Xk} explains the possibly highest range of variability in the area of the set of dependent variables {Y1,…, Ym}?
- Will the introduction of additional (new) variables independent or dependent on the analyzed sets affect the value of the total variance (increase its value)?
- Which variables from the set of variables {X1,…, Xk} jointly explain the largest range of variation within the group of variables {Y1, Y2,…, Ym}?

The canonical correlation allows assessing whether variables from one data set can be used to predict another set of data by finding such a linear combination of variables from the first set so that it correlates as strongly as possible with variables from the second set. It boils down to finding the vectors of coefficients (a1, a2… ap) and (b1, b2… bq) such that the correlation cor(a'X; b'Y) would be as large as possible. Thanks to this, new variables are created, so-called hidden or canonical variables, i.e. u1 = a'X; v1 = b'Y, which explains a significant part of the variability between the analyzed sets.

In the next steps, hidden variables are found, i.e. ui = aiX; vi = biY with the largest correlations cor(ui; vi). The designated hidden variables are a measure of the correlation between the sets {X1,…, Xk} and {Y1,…, Ym} and their weighted sums.

In the canonical analysis, the selection of canonical variability coefficients for particular variables is crucial in such a way that the two weighted sums (a1X1 + a2X2 +… + apXk and b1Y1 + b2Y2 +… + bqYm) were maximally correlated. The structure of variables and their specific contribution is reflected precisely by canonical weights. The higher the value (positive or negative) of the scale, the larger the contribution of a given variable to the canonical variable. Canonical weights are often given for standardized variables (mean = 0, standard deviation = 1), which facilitates their evaluation (comparison). These weights can be interpreted as beta coefficients in multiple regression. Correlations between hidden variables are called canonical correlation. A low canonical correlation or lack of it may indicate a wrong model or lack of connections between the analyzed two sets of variables. The interpretation of canonical correlations cannot be interpreted in the same way as Pearson's correlation. In the case of canonical analysis, this is only an auxiliary coefficient showing the extent to which the pair of weighted sums have been linked (correlated).

In the canonical analysis, correlations between canonical variables and variables in each group of variables are also determined. They are the so-called canonical factor loadings. The greater the value of the factor charge, the larger the contribution of the canonical variable. The value of factor charges elevated to the square represents the proportion of the variance of a given variable explained by the canonical variable (interpretation similar to the coefficient of determination). The average calculated for a given canonical variable after all variables give information about how many percent of the variance is explained by the given canonical variable on average. This is called variance on fractional variance deposition on canonical variates.

In a canonical analysis, there is a parameter called redundancy. It is calculated by increasing the canonical correlation to the square and multiplying by the variances calculated for the set of independent variables {X1,…, Xk} For example, if the redundancy of the first canonical variable {X1,…, Xk} is 0.76, it means that this variable explains 76% of the variation in the set {Y1,…, Ym}.

Summing up, the canonical analysis consists in:

- finding hidden variables.
- canonical variables that are not correlated with each other, which give information about specific variability in two data sets.
- calculation of canonical weights describing the pure contribution of each variable to the canonical variable.
- calculation of factor loads determining the correlation of each variable with the canonical variable.
- calculation of the redundancy (by calculating the variance) that tells how much the average variance of one set is explained by the given canonical variable by means of the variables of the second set of data.

The classic canonical correlation has many limitations including:

- variable distributions should be multidimensional normal.
- variables in collections should not be collinear (data redundancy prevents reliable presentation of results). This dependence allows capturing the correlation matrix.
- the outlying points should be rejected because the canonical analysis is very sensitive to them.
- it is recommended to use at least 20 times as many elements of the sample group as variables for the analysis.

#### Partial Least Squares regression (PLS)

Partial Least Squares Regression (PLS), like canonical correlation, is used to estimate the relationship between lipidomic and transcriptomic data. The practical difference between the application of canonical analysis and the partial Least Squares Regression is mainly based on other initial assumptions. No rejection of correlated dependent and independent variables is required, and the number of observations may be smaller than the number of predictors. Therefore, PLS is used in the exploratory analysis to choose convenient predictors and to identify outliers.

The PLS method (often referred to as "latent mapping") combines features of principal component analysis and multiple regression. First, it extracts a set of latent factors that explain as many covariances as possible between independent and dependent variables (a covariance matrix is created). Then, the values of dependent variables are predicted using the decomposition of independent variables. The PLS method is particularly useful when predictors are highly correlated (variables X) or when the number of variables is greater than the number of observations. PLS can be used in situations where the use of traditional multidimensional methods is severely limited. PLS is the least restrictive extension of the linear multiple regression model, which is also the basis for many other multidimensional methods. PLS can be used in situations where the use of traditional multidimensional methods are not effective.

The constraints that are not imposed by the PLS method are: (1) factors reflecting variables from the set Y and X are extracted based on the matrix Y'Y and X'X, respectively, and not based on the matrix of mixed products concerning both the variables from the set Y and from the set X and (2) the number of prognostic functions never exceeds the minimum from the number of variables from the set Y and X. In the case of PLS, prognostic functions are represented by factors determined based on the matrix Y'XX'Y. The number of such prognostic functions that can be determined usually exceeds the maximum from the number of Y and X variables. PLS can be used as a mining analysis tool to select prediction variables (it finds sets of independent variables) and to find outliers before applying classical linear regression.

The purpose of PLS (partial least-squares regression) is to build a linear model in the form Y = XB + E where:

Y—matrix of variable relationships with dimensions n (number of cases) per m (number of variables), X—matrix of independent variables with dimensions n (number of cases) per k (number of variables), B—matrix of regression coefficients with dimensions k on m, E is a random component of a model with dimensions such as the Y matrix.

In the PLS procedure, factorial values are calculated as linear combinations of non-convergent variables (predictors) in such a way that there is no correlation between variables of factor values used in the prognostic regression model.

An important step in the PLS analysis is to check the significance of latent variables with cross-validation. For example, take a set of response data (dependent variables Y) and predictors (independent variables X), which can be in part significantly correlated with each other. For such data, the matrix of factor values T = XW (W—the matrix of weights) is determined and then the linear regression model Y = TQ + E (Q—matrix of regression coefficients (loads)) can be used. The weight matrix W reflects the covariance structure between independent variables (predictors) and dependent variables (responses) in that it maximizes the covariance between dependent variables and the corresponding factor values. Next, the least-squares method determines the Y-regression against T for determining the Q matrix with charges (weights) for Y such that Y = TQ + E. When calculating the values of charges, the equation Y = XB + E (B = WQ) is obtained, which serves as a prognostic regression model.

In the PLS procedure, a matrix of factor charges P is also obtained, which allows obtaining factor model X = TP + F (F—unexplained part of X results). The regression of partial least squares is a very efficient analytical method of intra-dimensional data, however, in the case of biological data, it is not always sufficiently effective to draw biological conclusions from the point of view.

The OmicsON library includes a PLS calculation library that enumerates all of the above operations. In addition to basic information on the percentage of explained variations, the procedure provides the values of individual factor matrices, weights and regression coefficients, residues and model fit results necessary to assess the correctness of the predictive model and the correct interpretation of the final results. The cross-validation procedure for prediction of the variable matching error i.e. RMSEP (root mean squared error of prediction), MSEP (mean squared error of prediction) or R2 (coefficient of multiple determination) is applied in the PLS library. The error evaluation allows for the proper selection of variables in the model so that they best describe the analyzed process.

### Biological interpretation

Reducing the dimensions of two data sets and obtaining hidden variables (CV in the case of CCA and LV in the case of PLS) allows the observation of relationships between two sets of variables, transcriptomic and metabolic in this case. Relating gene expression with fatty acids pave the way for the biological interpretation of such a dependence. Thanks to this, often unobvious relationships of two sets of variables explain or show the direction of the course of the analyzed biochemical processes.

## Results

As an example application of the OmicsON library, sets of fatty acids and gene transcription from a collection of murine nutrigenomics study was used [24]. The population comprises mice nurtured in five different diet regimes, with 40 individuals in total. Based on the analysis of hepatic samples (four biological replicates at three-time points each), it comprises gene expression data of 120 selected genes potentially involved in lipid metabolism and concentrations of 21 fatty acids. This is not a large data set, but sufficient to demonstrate the functionality of the OmicsON library.

The purpose of the analysis was to check (with CCA and PLS) between which variables, i.e. genes (transcripts) versus fatty acids (metabolites) there is an association.

### Gene mapping

In the first step, the gene mapping procedure was performed based on knowledge from Reactome [25]. For each class of fatty acids for a given list of small molecule ChEBI identifiers [26], associated reactions in the Reactome database were found. All genes found in this set of reactions define a group of genes initially associated with a given class of fatty acids. In the case where ChEBI's id is presented in ChEBI2Reactome.txt (automatically taken by the OmicsON library from the Reactome database) file then the mapping is taken from that file. When we cannot find that our ChEBI's id is presented in this file, then we search the ChEBI's ontology tree. We use a function which favoring children nodes, the closest child is taken as representative. Of course, the used child node should be presented in the mentioned mapping file. In the case where no children are presented, then closest parent is taken. ChEBI's ids without any representation are not considered under further analysis.

In the next step, the list of genes was decorated by genes taken from the STRING database [27]. A set of genes from the previous step is used to build a query in the STRING database via the STRINGdb Bioconductor library [28]. This group of genes is expanded to include the closest neighbors of these genes found in the gene interaction network to find interacting proteins and associations between genes (Table 1).

**Table 1:** List of genes and fatty acids after functional grouping with REACTOME and STRING using.

lipids ID (CHEBI)	Genes symbols (HGNC)
28875,28875,73705, 17268,15756,28842, 35465,28716,16196, 36023,32425,36036, 17351,28661,72850, 15843,61205,61204, 27432,28364,28125	ACAT1,ACAT2,APOB,APOC3,APOE,CBS,CIDEA,CPT2,CYP27A1,CYP27B1,CYP8B1,FAS,GK,LDLR, LPIN2,LPIN3,LPL,MTHFR,PDK4,PEX11A,PLTP,PPARA,PPARD,PPARG,RARA,RXRA,UCP2,UCP3, VDR,VLDLR,DBI,ACACA,ACACB,SSX2IP,ADSSL1,ALDH3A1,ACOX1,PSMB10,ABCB11,BCL3, PRG4,CAR1,COX1,COX2,CYP24A1,CYP26A1,CYP2B10,CYP2B13,CYP2C29,CYP3A11,CYP4A10, CYP4A14,CYP7A1,FAT1,NR1H4,H6PD,G6PC,GLUL,GSTA,GSTM1,GSTP2,HMGCR,Il2,FABP1,ELOVL6,COL2A1,NR1H3,NR1H2,LPIN1,CPT1A,ACADM,ABCB1B,ABCB4,ABCC6,MTR,NR4A1,NR4A2,SLC10A1,SLC22A5,NRF1,ECI2,PON1,NR1I2,RARB,RXRG,THRSP,PTPN6,ST3GAL4,SERPINA1A,SCARB1,LY6D,TRA2B,AATF,TMPO,HADHB,CDKN1A,TFAP2A,APOA1,FOS,ABCC2,EIF2S3X,ABCA1,FABP6,BAAT,FABP2,NOS2,ABCB8

As a result, a subset of genes functionally related to a set of fatty acids was obtained.

### Autocorrelation in transcriptomic and lipidomic sets

Very often the number of genes (variables) is much larger than the number of samples (observations). Canonical correlation is sensitive to the number of observations and thus the calculation of CCA is not possible with original sizes sets of data. The transcriptomic (set of genes) and lipidomic (set of fatty acids) data of the sets were checked for internal correlations because performing the canonical correlation requires the removal of correlated predictors (independent, transcriptomic variables—X) and responses (independent lipidomic variables—Y) (supplementary material S1 Data and S2 Data). The correlation threshold is set arbitrarily and is closely related to the data type. Representatives (under the cutoff of internal correlation) were selected for further analysis (in supplement data), while the rest were taken into account only at the stage of biological interpretation (Table 2).

**Table 2:** List of genes and fatty acids after turning off correlated data within individual sets. R correlation coefficient cut-offs were assumed arbitrarily. It was for genes: r = 0.6 and for fatty acids: r = 0.7.

Genes symbols (HGNC)	lipids ID (CHEBI)
APOB, VLDLR, PSMB10, PRG4, CYP26A1, ECI2, NR1I2, RXRG, CDKN1A, APOA1, NOS2, ABCB8	73705, 17268, 15756, 28842, 36023, 32425, 36036, 28661, 61204, 27432, 28364, 28125

### Canonical correlation results

A properly prepared set of data was subjected to canonical correlation analysis, the essence of which is to find such linear combinations of variables in the analyzed sets that the correlation between them is as large as possible. In this case, the strength of the relationship between transcriptomic and lipidomic variables is very high and statistically significant for the first hidden variables. Obtained canonical correlations for the analyzed data were: CV1 = 0.967 (p = 3.23e-09), CV2 = 0.94 (p = 4.016e-05), CV3 = 0.86 (p = 0.013). Significance assessment calculated based on Bartlett's Chi-Squared test.

The OmicsON library allows obtaining also many detailed information when calculating canonical correlations such as Canonical Variate Coefficients, Structural Correlations (Loadings), Fractional Variance Deposition on Canonical Variates, Canonical Communalities (Fraction of Total Variance Explained for Each Variable, Within Sets), Canonical Variate Adequacies (Fraction of Total Variance Explained by Each CV, Within Sets), Redundancy Coefficients (Fraction of Total Variance Explained by Each CV, Across Sets).

The rest of the manuscript focuses on discussing the first hidden variable.

**Fig 2:**
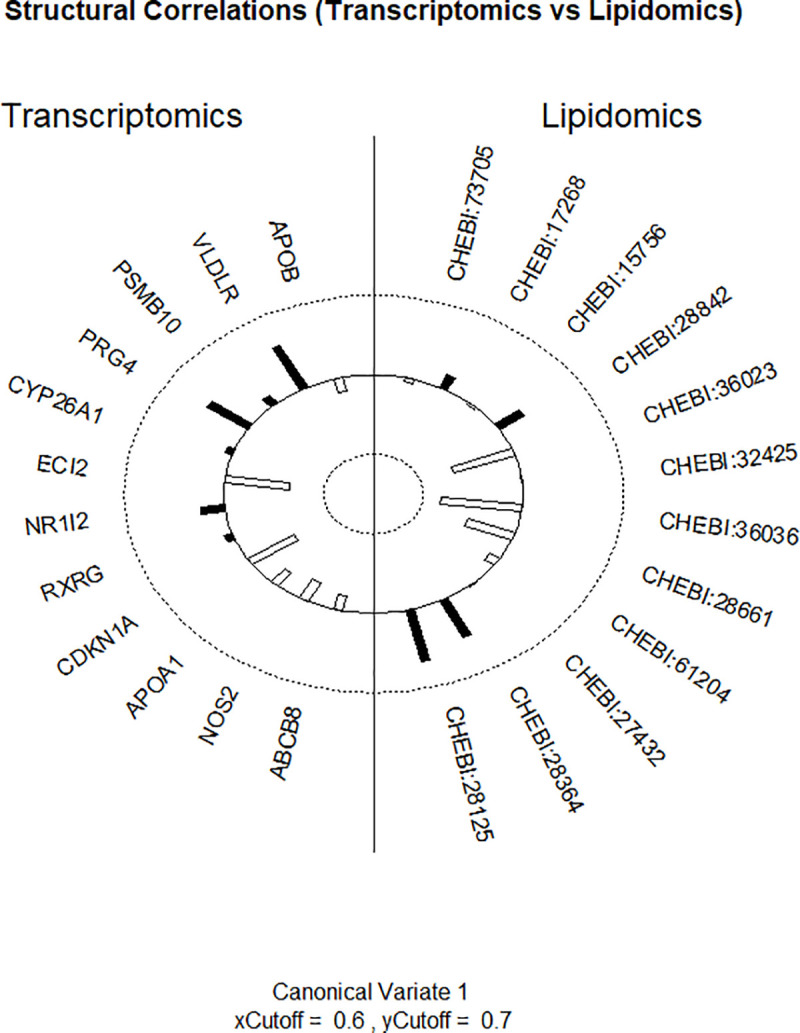
The canonical correlation results for one subset of transcriptomic and lipidomic data. Positive correlation represented by bars directed to the outside of the circle, and a negative correlation towards the inside. The height of the bars indicates the strength of the association.

The figure (helio plot) shows which of the lipids are correlated with gene expressions (Fig 2). The positive correlation represents the bars towards the outside of the inner circle, and the negative correlation visualizes the bars facing inside. The height of the bar indicates the height of the correlation coefficient. The higher the bar the higher the correlation, the lower the bar the lower correlation. The analysis results for the first subgroup show that the gene expression: very-low-density lipoprotein receptor (VLDRL) and proteoglycan 4 (PRG4) is positively correlated with docosahexaenoic acid (28125) and an icosapentaenoic acid (28364), as well as negatively correlated with icosatrienoic acid (36036), vaccenic acid (36023) and γ-linolenic acid (28661). The strength of correlation in these cases is significantly higher compared to other fatty acids and expression of genes. The high strength of a relationship is also observed for enoyl-CoA delta isomerase 2 (ECI2) and cyclin-dependent kinase inhibitor 1A (CDKN1A) with some fatty acids. The reduced gene expression level of ECI2 and CDKN1A correlates with high levels of fatty acids, i.e. docosahexaenoic acid (28125) and an icosapentaenoic acid (28364), and low concentrations of vaccenic acid (36023), γ-linolenic acid (28661).

### Partial least squares regression results

The partial least squares regression analysis (PLS) as an alternative method of analysis of the canonical correlation was performed. The purpose of this analysis was to find a linear combination of variables from the transcriptomic and lipidomic data sets with the regression method to find the relationships (contributions) of the individual variables with each other. The PLS procedure also allows grouping data sets taking into account the contribution of variables to the description of the model.

The biological interpretation of the effect of all fatty acids on all genes is difficult from an interpretative point of view, hence the set obtained after the functional analysis was included in the PLS procedure. Several "percentage variances explained" for latent variables (LV) were calculated based on the given data sets. The percentage of explained variation allows assessing whether given sets of data satisfactorily describe relationships between variables. For a ten latent variables the following percentage explained variabilities were: LV1 = 32.57%, LV2 = 51.55%, LV3 = 63.77%, LV4 = 68.84%, LV5 = 75.63%, LV6 = 78.32%, LV7 = 80.91%, LV8 = 83.10%, LV9 = 84.79%, LV10 = 86.00%. The second latent variable will explain over 50% of the variability of the analyzed data sets, and the sixth close to 80%.

**Fig 3:**
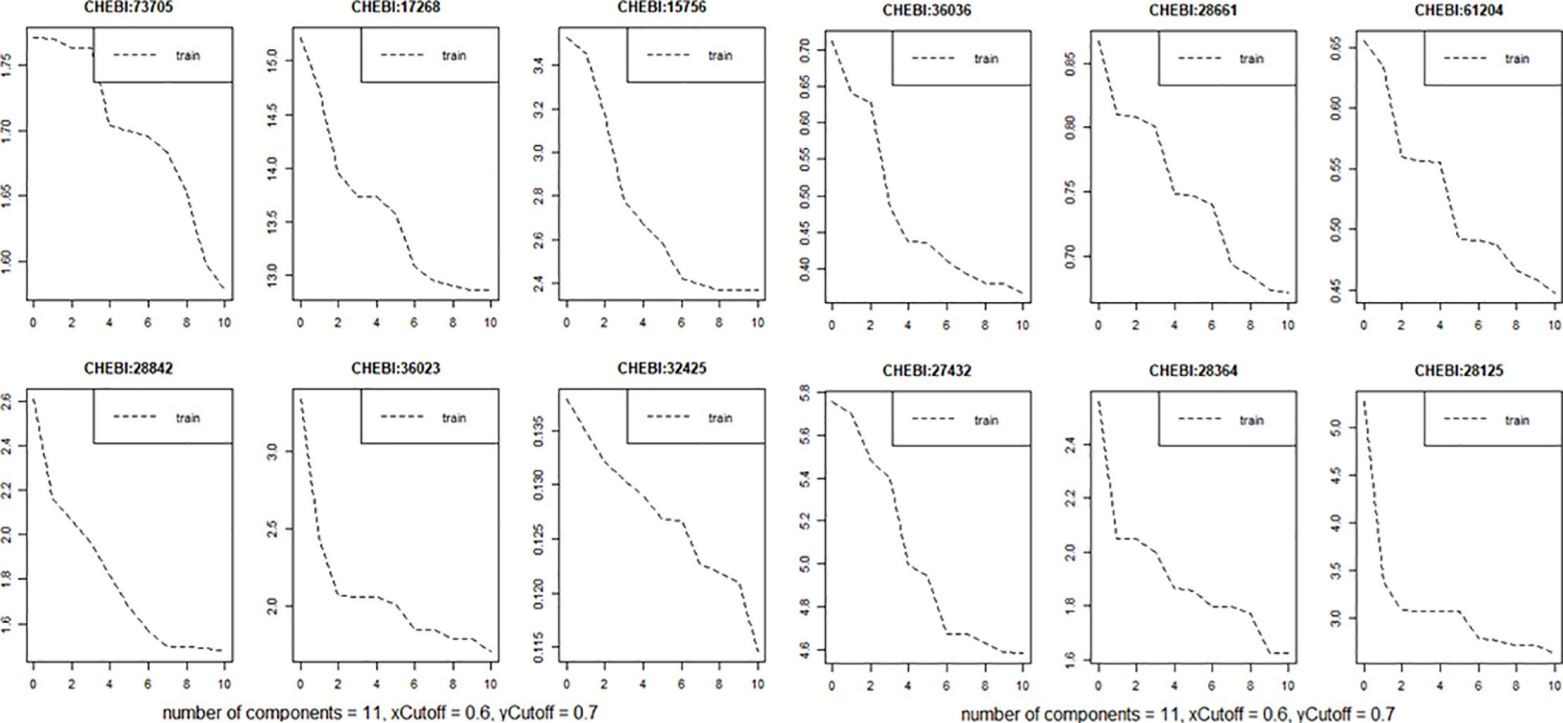
Cross-validated RMSEP curves for the fatty acid variables. The smaller the prediction error, the better the given variable is suitable for the predictive model.

In addition to “percentage explained variation”, several other statistics were calculated, including regression coefficients, scores, loadings, loading.weight, Yscores, Yloadings, projection. They allow assessing the contribution (weight) of individual transcriptomic and lipidomic variables to the model. Having information about the input of individual variables, it can be build models used for prediction. It enables the selection of variables based on the determination of the prediction error e.g. root mean squared error of prediction (RMSEP) and the selection of a latent variable for interpretation in the case of a specific variable. As an example, the results of RMSEP for fatty acids are presented (Fig 3). The interpretation of the contribution of individual fatty acid variables can be interpreted based on latent variables for which the prediction error is the smallest. The results obtained show that at the level of the first latent variable, the largest contribution to the linear model of the relationship of fatty acids and genes have fatty acids with the CHEBI identifiers: 61204, 36036, 28661, 32325, 73705, 28842, 28364, 28125 (Fig 3). The RMSEP calculated for them is the smallest.

## Discussion

The OmicsON library is a tool for the integration of transcriptomic and metabolomic data. The procedures implemented in the library allow for gene mapping based on databases, i.e. Reactom and String, and for statistical analyzes, i.e. canonical analysis (CCA) and Partial least squares regression (PLS). This approach provides information on the relationship between the transcription level of specific genes and the level of metabolites. Correlating this information can significantly help in the interpretation of biological processes analyzed.

The OmicsON library has many advantages, but also several limitations, both from biological data and statistical methods. The results obtained based on the OmicsON library could be almost fully trusted when you could be sure that the data sets, such as Reactome or String are complete. Meanwhile, it is known that these databases are constantly replenished, but they still do not fully provide a complete description of the relationship between molecules. For some organisms, there are more descriptions and relatively few for others. The library gives satisfactory results in the case of:

- studying the data of well-annotated species.
- access to reliable molecular networks to achieve a functional relationship between individuals.
- a well-functioning internet network, as some procedures require communication with external databases
- grouping of metabolomic and transcriptomic data in such a way that canonical correlation (CCA) can be used since CCA cannot be used for a large number of observations and a small number of test samples.

Another disadvantage that can be encountered is the problem with the availability of two sets of data (e.g. transcriptomic and metabolomic) from one organism. Available public repositories collect and provide data sets usually for one type of data, e.g. only transcriptomic or only metabolomic data. To demonstrate all the functionalities of the OmicsON library, the authors used (the only data set available at that time) that concerned the mouse. The results of calculations were demonstrated based on Reactom and String datasets for humans (without narrowing to specific tissues) because the most complete data set is currently for human. This is an inaccuracy, but we would like to emphasize that the goal was to demonstrate the full functionality and capabilities of the OmicsON library and not a detailed interpretation of concrete the biological processes.

## Availability and future directions

OmicsON is available from https://github.com/cmujzbit/OmicsON.

In omics-based studies, small support of biological samples is often observed. This is associated with the high costs of wet laboratory and sequencing as well as the difficult availability of clinical samples. Researchers address this problem by selecting appropriate computational methods. Classical statistics methods are not efficient in such cases, therefore the research challenge is to use appropriate extensions or combinations of these mathematical tools. This opens new possibilities for an analysis of a large data sets with a small number of observations.

One of the most effective ways to achieve improved results is to combine basic statistical techniques (also called gold standard methods) with innovative approaches like regulated canonical analysis (rCCA) [29] or regularization and grouping data by GCA method [30][31]. This ensures effective analysis of data in which the number of variables significantly exceeds the number of observations [29,32][12].

In the future, the OmicsON will be improved through the implementation of univariate models, including multiple testing correction strategies, dimension reduction techniques [30], and variable selection models [30] [33].

## Supporting information

S1 Table List of genes and lipids. CleanData_06_08—Full experiment data with correlation's threshold 0.6 for X and 0.8 for Y, CleanData_06_07- Full experiment data with correlation's threshold 0.6 for X and 0.7 for Y, Reactome_Ensemble_06_07- Experiment data cutoff to Reactome pathways base on Ensemble IDs with correlation's threshold 0.6 for X and 0.7 for Y, Reactome_UniProt_08_09—Experiment data cutoff to Reactome pathways base on Ensemble IDs with correlation's threshold 0.8 for X and 0.9 for Y, String_Expand_Ensemble_06_07- Experiment data cutoff to String relations base on Ensemble IDs with correlation's threshold 0.6 for X and 0.7 for Y.
(DOCX)

S1 Data (TXT)

S2 Data (TXT)
