# Supplementary material for: OmicsON – Integration of omics data with molecular networks and statistical procedures
Source: PLoS One. 2020 Jul 29;15(7):e0235398. doi: 10.1371/journal.pone.0235398 (PMC7390260; doi:10.1371/journal.pone.0235398)
Supplement: S1 Table — CleanData_06_08—Full experiment data with correlation's threshold 0.6 for X and 0.8 for Y, CleanData_06_07- Full experiment data with correlation's threshold 0.6 for X and 0.7 for Y, Reactome_Ensemble_06_07- Experiment data cutoff to Reactome pathways base on Ensemble IDs with correlation's threshold 0.6 for X and 0.7 for Y, Reactome_UniProt_08_09—Experiment data cutoff to Reactome pathways base on Ensemble IDs with correlation's threshold 0.8 for X and 0.9 for Y, String_Expand_Ensemble_06_07- Experiment data cutoff to String relations base on Ensemble IDs with correlation's threshold 0.6 for X and 0.7 for Y. (DOCX) [file pone.0235398.s001.docx]

Table 1. List of genes and lipids.
CleanData_06_08 - Full experiment data with  correlation's threshold 0.6 for X and 0.8 for Y, 
CleanData_06_07- Full experiment data with  correlation's threshold 0.6 for X and 0.7 for Y,
Reactome_Ensemble_06_07- Experiment data cutoff to Reactome pathways base on Ensemble IDs with correlation's threshold 0.6 for X and 0.7 for Y, 
Reactome_UniProt_08_09 - Experiment data cutoff to Reactome pathways base on Ensemble IDs with correlation's threshold 0.8 for X and 0.9 for Y, 
String_Expand_Ensemble_06_07- Experiment data cutoff to String relations base on Ensemble IDs with correlation's threshold 0.6 for X and 0.7 for Y,

| **Group** | **genes (HGNC)** | **lipids (CHEBI)** |
| --- | --- | --- |
| CleanData_06_08 | APOB, VLDLR, PSMB10, PRG4, CYP26A1, ECI2, NR1I2, RXRG, CDKN1A, APOA1, NOS2, ABCB8 | 73705, 17268, 15756, 28842, 36023, 32425, 36036, 28661, 61204, 27432, 28364, 28125 |
| CleanData_06_07 | APOB, VLDLR, PSMB10, PRG4, CYP26A1, ECI2, NR1I2, RXRG, CDKN1A, APOA1, NOS2, ABCB8 | 73705, 17268, 15756, 28842, 36023, 32425, 36036, 28661, 61204, 27432, 28364, 28125 |
| Reactome_Ensemble_06_07 | APOB, APOC3, APOE, CPT2, CYP8B1, LDLR, LPL, PEX11A, PLTP, PPARA, PPARG, RXRA, UCP2, UCP3, DBI, ACACA, ACACB, ACOX1, CYP7A1, NR1H4, HMGCR, FABP1, ELOVL6, NR1H3, NR1H2, CPT1A, ACADM, ABCB4, NRF1, ECI2, PON1, SCARB1, APOA1, ABCA1 | 28875, 73705, 17268, 15756, 28842, 28716, 16196, 36036, 17351, 28661, 72850, 15843, 61205, 61204, 27432, 28364, 28125 |
| Reactome_UniProt_08_09 | APOB, APOC3, CPT2, CYP8B1, PEX11A, PPARA, RXRA, ACACB, CYP7A1, NR1H4, FABP1, ELOVL6, NR1H3, NR1H2, CPT1A, ACADM, PON1, APOA1, ABCA1 | 73705, 17268, 15756, 28842, 28716, 16196, 36036, 17351, 28661, 72850, 15843, 61205, 61204, 27432, 28364, 28125 |
| String_Expand_Ensemble_06_07 | APOB, VLDLR, PSMB10, PRG4, CYP26A1, ECI2, NR1I2, RXRG, TMPO, CDKN1A, APOA1, NOS2, ABCB8 | 73705, 17268, 15756, 28842, 16196, 36036, 2866,  61204, 27432, 28364, 28125 |
